# Hepatocyte Proliferation/Growth Arrest Balance in the Liver of Mice during *E. multilocularis* Infection: A Coordinated 3-Stage Course

**DOI:** 10.1371/journal.pone.0030127

**Published:** 2012-01-10

**Authors:** Chuanshan Zhang, Junhua Wang, Guodong Lü, Jing Li, Xiaomei Lu, Georges Mantion, Dominique A. Vuitton, Hao Wen, Renyong Lin

**Affiliations:** 1 State Key Laboratory Incubation Base of Major Diseases in Xinjiang and Xinjiang Key Laboratory of Echinococcosis, First Affiliated Hospital of Xinjiang Medical University, Urumqi, Xinjiang, China; 2 World Health Organization-Collaborating Centre for the Prevention and Treatment of Human Echinococcosis, Department of Digestive Surgery; Jean Minjoz Hospital, University of Franche-Comté and University Hospital, Besançon, France; 3 Research Unit EA 3181 “Epithelial Carcinogenesis: Predictive and Prognostic Factors,” University of Franche-Comté, Besançon, France; The George Washington University Medical Center, United States of America

## Abstract

**Background:**

Alveolar echinococcosis (AE) is characterized by the tumor-like growth of *Echinococcus* (*E.*) *multilocularis*. Very little is known on the influence of helminth parasites which develop in the liver on the proliferation/growth arrest metabolic pathways in the hepatocytes of the infected liver over the various stages of infection.

**Methodology/Principal Findings:**

Using Western blot analysis, qPCR and immunohistochemistry, we measured the levels of MAPKs activation, Cyclins, PCNA, Gadd45β, Gadd45γ, p53 and p21 expression in the murine AE model, from day 2 to 360 post-infection. Within the early (day 2–60) and middle (day60–180) stages, CyclinB1 and CyclinD1 gene expression increased up to day30 and then returned to control level after day60; Gadd45β, CyclinA and PCNA increased all over the period; ERK1/2 was permanently activated. Meanwhile, p53, p21 and Gadd45γ gene expression, and caspase 3 activation, gradually increased in a time-dependent manner. In the late stage (day180–360), p53, p21 and Gadd45γ gene expression were significantly higher in infected mice; JNK and caspase 3 were activated. TUNEL analysis showed apoptosis of hepatocytes. No significant change in CyclinE, p53 mRNA and p-p38 expression were observed at any time.

**Conclusions:**

Our data support the concept of a sequential activation of metabolic pathways which 1) would first favor parasitic, liver and immune cell proliferation and survival, and thus promote metacestode fertility and tolerance by the host, and 2) would then favor liver damage/apoptosis, impairment in protein synthesis and xenobiotic metabolism, as well as promote immune deficiency, and thus contribute to the dissemination of the protoscoleces after metacestode fertility has been acquired. These findings give a rational explanation to the clinical observations of hepatomegaly and of unexpected survival of AE patients after major hepatic resections, and of chronic liver injury, necrosis and of hepatic failure at an advanced stage and in experimental animals.

## Introduction

The larval stage of the fox-tapeworm *Echinococcus* (*E.*) *multilocularis* is the causative agent of alveolar echinococcosis (AE), one of the most dangerous parasitic disease of the northern hemisphere [1]. AE is characterized by an infiltrative, destructive and tumor-like growth of the *E. multilocularis* metacestode, usually affecting the liver of natural intermediate hosts such as small rodents or the human liver [2]. Clinical manifestations are results of both a slow but continuous asexual proliferation of the metacestode and an intense infiltration by macrophages, T lymphocytes, and fibroblasts/myofibroblasts around the parasite, eventually leading to fibrosis and necrosis [3], [4], [5], [6]. Clinical symptoms usually appear many years after the first contact with the parasite eggs (oncospheres); progression of the lesions is very slow and the observed complications and liver dysfunction are the result of a complex and often latent sequence of events. In the experimental model of secondary *E. multilocularis* metacestode infection, which well mimics the natural infection [7], [8], according to its clinical course AE is divided into 1) an early stage with tumor-like growth of the metacestode and mild hepatic enlargement, 2) a middle stage with invasive parasitic lesions and progressive hepatomegaly and 3) an advanced/terminal stage (also called “late stage”) associated with invasion of other organs and/or metastases, fibrosis of the lesions and cholestasis, which may cause secondary liver cirrhosis with subsequent portal hypertension and eventually impaired liver function [9]. We and others have shown in previous studies that these clinical changes were accompanied by a typical course of cytokine production, with, sequentially 1) a Th1 profile followed by 2) a combined Th1 and Th2 profile, also characterized by a markedly increased production of IL-10 [10], and finally 3) a decrease in all types of cytokines associated with a deep impairment of the immune response [11], [12]. Changes with time in a variety of other components/enzymes involved in the immune response such as chemokines [13], proteins of the acute inflammatory phase [14], and nitric oxide synthase [15] have also been shown. However, despite the presence of well-known clinical symptoms (hepatomegaly, liver necrosis) which evoked such influence in patients with AE, until recently, little was known on the influence of the metacestode on the hepatocytes of the surrounding liver parenchyma.

The orderly progression of cells through the phases of the cell cycle is governed by the sequential assembly and activation of holoenzyme complexes [16]. The Mitogen-Activated Protein Kinase (MAPK) pathway and cell cycle regulatory proteins, including Cyclins, Cyclin-dependent kinases (Cdks), Cyclin-dependent kinase inhibitor 1α (Cdkn1α or p21), growth arrest and DNA damage-inducible 45(Gadd45α, Gadd45β and Gadd45γ), participate in the regulation of cell cycle progression [17], [18], [19], [20]. Importantly, CyclinD1, a regulator of cellular proliferation, is itself regulated by ERK1/2 [21], [22]. The Cip/Kip family member, p21 was shown to inhibit cell proliferation and activities of several Cyclin-Cdk complexes *in vitro* [23], [24]. Transcriptional regulation of the p21 gene is controlled by the tumor suppressor protein p53 acting on the p53 responsive element in the distal region of the p21 promoter in response to intracellular signals such as DNA damage [25], [26]. Furthermore, Gadd45γ is also a p53-regulated human gene, which interacts with PCNA, a normal component of multiple quaternary complexes, including the Cycling Cdks and the Cdk inhibitor p21, which play a central role in DNA repair, growth suppression and apoptosis [27], [28], [29]. In addition, the JNK and p38 cascades appear to be pro-apoptotic. Their activation, via MTK1/MEKK4, is mediated by Gadd45γ as was shown in response to various external stresses including bacterial infection, hyperosmolarity, and UV irradiation; these cascades also appear to be closely related to cell death [30], [31], [32].

Our previous study, using western blot technique, has shown that metabolic pathways involved in liver proliferation and growth arrest and especially the MAPKs system were specifically induced by *E. multilocularis* growth. We observed that ERK1/2 and p38 were activated in the liver of AE patients and that ERK, JNK and p38 were activated in rat primary hepatocytes during exposure to *E. multilocularis* vesicle fluid (EmF) or *E. multilocularis* axenic culture supernatant (EmCM) *in vitro* [33]. Furthermore, using microarray and qPCR technique, we followed the time-course of *E. multilocularis* infection from day30 to 180 after intrahepatic injection of metacestode for the expression of genes involved in the inflammatory/immune response as well as numerous metabolic pathways specific to the liver. We found an increased expression of Gadd45β (2.19 fold at day90; 4.49 fold at day180), Gadd45γ (3.98 fold at day60; 4.92 fold at day90 and 21.94 fold at day180) and p21 (5.60 fold at day60; 4.42 fold at day180) in the middle and late stages of *E*. *multilocularis* infection in mice [34]. Gadd45β gene was originally characterized as a primary responder in myeloid differentiation induced by IL-6 [35]. More recent studies have shown that Gadd45β, unlike two other homologs (Gadd45α and γ), plays an anti-apoptotic role and is activated by TNF-α *v*ia NFκB [19]. Thus, induction of Gadd45β coincides with the entry into an active cell cycle; its action might be to protect hepatocytes from apoptosis in the middle stage. Then induction of p21 and Gadd45γ coincides with the entry into an inhibitory phase regarding cell proliferation; its action might be to promote hepatocyte growth arrest and/or apoptosis in the late stage. However, the underlying mechanisms for the involvement of the host liver MAPK signaling pathways, and of cell cycle regulated proteins such as Cyclins, Gadd45β, p53, p21 and Gadd45γ in the progression of *E. multilocularis*-infected mice *in vivo* are unknown to date. Their contribution to the hepatocyte proliferation and growth arrest process which appears to accompany *E. multilocularis* metacestode development is also ignored.

The aims of the present study were thus, in the secondary experimental murine model of AE, 1) to explore the influence of *E. multilocularis* metacestode on components of cell cycle regulation which characterize the host's hepatic proliferation in the liver of mice infected with *E. multilocularis* over a time period of 1yr, i.e. from the date of *E. multilocularis* inoculation to the very late stage of infection; 2) to simultaneously explore the activation of inhibitory proteins involved in growth arrest/apoptosis metabolic pathways during the 3 stages of infection. For these purposes, we measured the levels of ERK1/2, JNK, p38 activation, Cyclins, PCNA, Gadd45β, Gadd45γ, p53 and p21 by western blot and qPCR and, using immunohistochemistry we studied the same components in relation to the pathological changes in the liver, both in the infection site and in the neighboring liver parenchyma where proteins and mRNAs were measured.

## Results

### Hepatic injury induced by *E. multilocularis*

**Figure 1:**
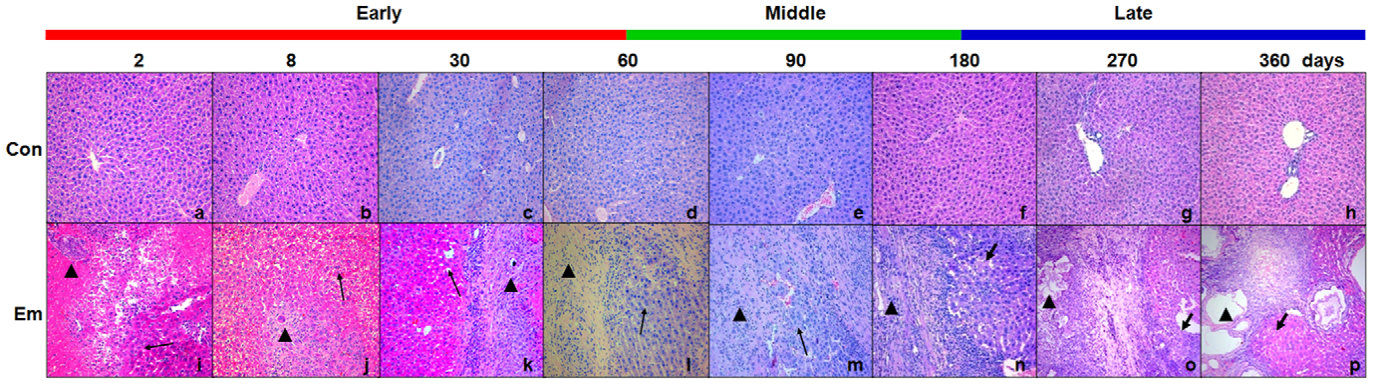
Representative histopathology of mice liver during *E. multilocularis* infection. (a–h): No morphological changes were observed in the liver of control mice. (i–p): Proliferating hepatocytes close to the parasitic lesions were observed from days 2 to 180 (*thin arrow*) and some coagulation necrosis areas (*thick arrow*) close to the parasitic lesions were observed from days 180 to 360 in the liver from *E. multilocularis* infected mice. The *arrowheads* indicate the parasitic lesions in the liver of infected mice (“lesion” row); the lesions had the typical aspect of *E. multilocularis* germinal layer and laminated layer, surrounded by a periparasitic cell infiltrate composed mostly, from the center to the periphery, of macrophages and fibroblasts/myofibroblasts, and lymphocytes. Final magnification, 200×. con, control, non-infected mice; Em, *E. multilocularis* infected mice.

As shown in Figure 1, from day30 to 180 post-infection, the typical pathological changes which characterize *E. multilocularis* lesions were observed in the liver of the mice infected with *E. multilocularis*; at days 2 and 8, only infiltrating lymphocytes, surrounding *E. multilocularis* inoculum, could be observed; at days 270 and 360 post-infection, the pseudo-tumor parasitic mass included many vesicular and/or cystic structures embedded within thick fibrous tissue, the periparasitic area was composed of inflammatory fibrous tissue and necrotic areas and mixed with small granulomatous nodules [34]. The hepatic parenchyma close to the lesions was progressively invaded by fibrous connective tissue septa, and solitary islands of hepatic tissue were observed. At the same time points, no hepatic injury was observed in the mice of the control groups (Figure 1).

### Activation of MAPKs in the liver of mice during *E. multilocularis* infection

**Figure 2:**
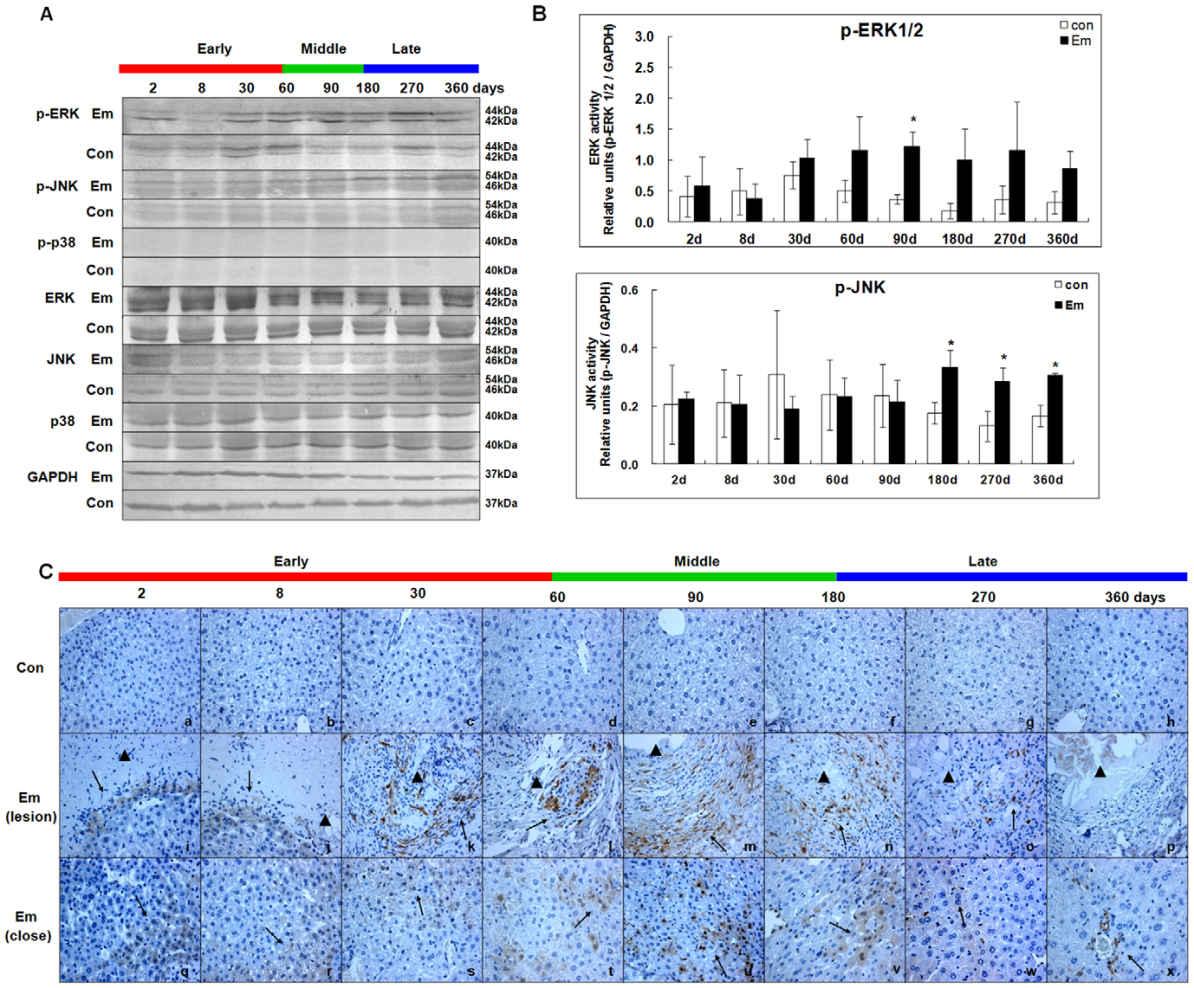
Activation of MAPKs in the liver of mice during *E. multilocularis* infection. Western blot analyses were performed on lysates from liver samples with antibodies that recognize phosphorylated (p-) and total ERK1/2, JNK and p38 respectively (A). Relative amount of phosphorylated and total ERK1/2, JNK and p38 was calculated from semi-quantitative analysis of the Western blots using densitometry (B). Histo-immunochemical analysis of p-ERK1/2 expression was performed on tissue samples: p-ERK1/2 expression was observed in infiltrating lymphocytes (lesion) and fibroblast-like cells (lesion) but also in hepatic cells (close to lesion) in the liver from *E. multilocularis* infected mice (*arrow*) (C). The *arrowheads* indicate the parasitic lesions in the liver of infected mice (“lesion” row). Final magnification, 400×. ^*^ *P*<0.05 versus control. con, control, non-infected mice; Em, *E. multilocularis* infected mice; AU: arbitrary units; lesion: *E. multilocularis* metacestode and surrounding immune infiltrate; close: liver parenchyma close to *E. multilocularis* lesion.

As shown in Figure 2A and 2B, western blotting showed increased ERK1/2 phosphorylation (p-ERK1/2) from day2 (∼1.40-fold) to 360(∼2.78-fold); it peaked at day180 (∼5.69-fold); there was a significant difference between *E. multilocularis* infected and non-infected mice at day90 post-infection (*P* < 0.05), and increased JNK phosphorylation (p-JNK) from day180 (∼1.89-fold) to 360 (∼1.85-fold); it peaked at day270 (∼2.19-fold) post-infection; it was significantly different between *E. multilocularis*-infected and non-infected mice at all time points since day180 (*P* < 0.05). Interestingly, no phosphorylated-p38 (p-p38) was observed in liver during the course of infection. As shown in Figure 2C, in the lesion area, p-ERK1/2 was expressed in a few hepatocytes at days 2 and 8 while there was no positive staining in the inflammatory response zone. At days 30 and 60 the intensity of the immunostaining increased, showing p-ERK1/2 localization in infiltrating lymphocytes and fibroblast-like cells. At days 90, 180 and 270, the staining was more intense in the infiltrating lymphocytes, and then decreased at day360. Within the liver parenchyma close to the lesion and peri-parasitic infiltrate, p-ERK1/2 was observed in hepatocytes from day2 to 60. At days 90 and 180, the number of hepatocytes which expressed p-ERK1/2 progressively increased, with staining intensities from “weak” to “moderate”; then p-ERK1/2 expression in hepatocytes decreased at day360. No liver cell positive for p-ERK1/2 immunostaining were observed in the liver of non-infected mice.

### Expression of Gadd45β in the liver of mice during *E. multilocularis* infection

**Figure 3:**
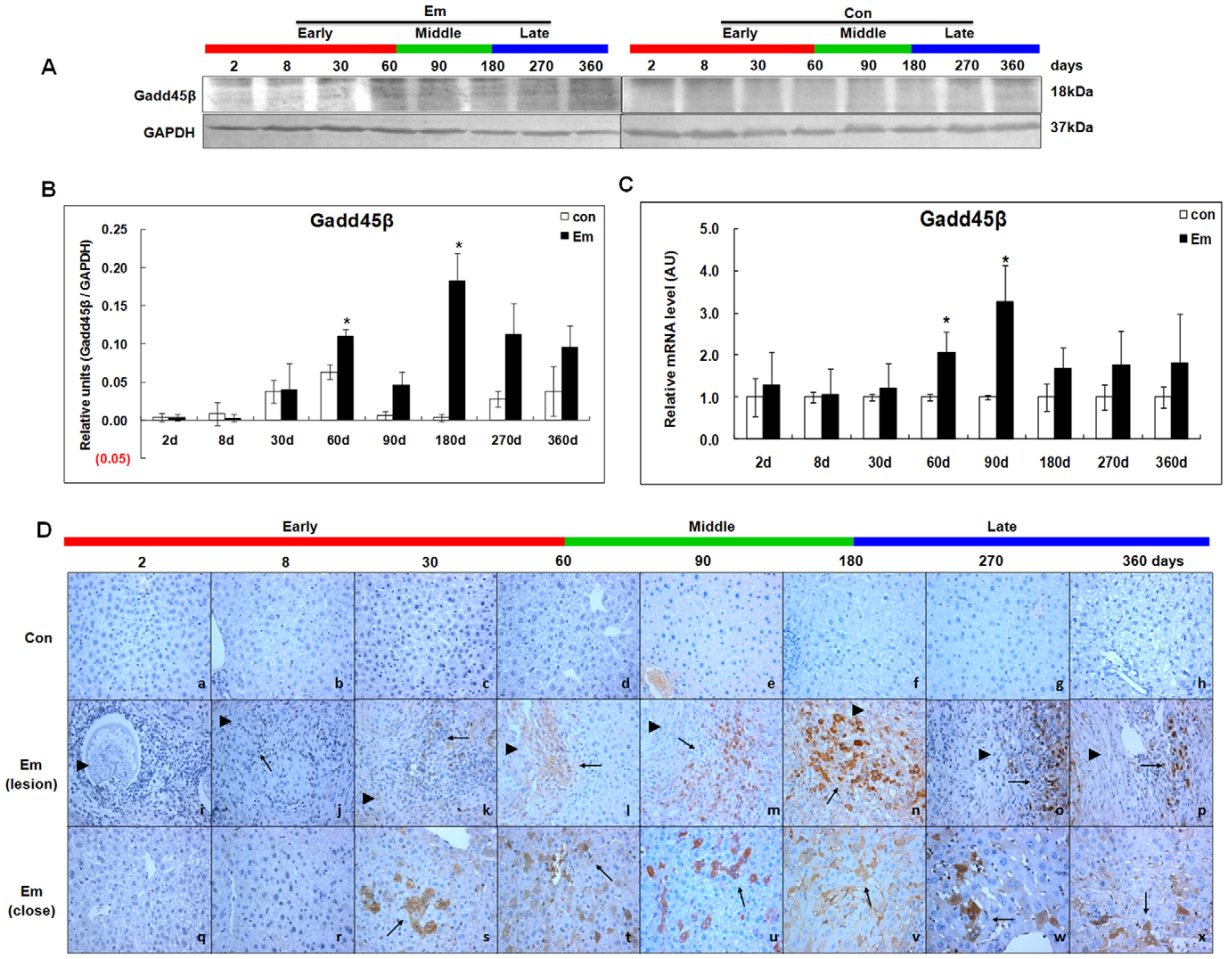
Gadd45β expression in the liver of mice during *E. multilocularis* infection. Western blot analyses were performed on lysates from liver samples with antibodies that recognize Gadd45β (A). Relative amount of Gadd45β expression was calculated from semi-quantitative analysis of the Western blots using densitometry (B). Gadd45β mRNA expression was measured by qPCR (C). Histo-immunochemical analysis of Gadd45β expression was performed on tissue samples: Gadd45β expression was observed in infiltrating lymphocytes (lesion) but also in hepatic cells (close to lesion) in the liver from *E. multilocularis* infected mice by histo-immunochemical analysis (*arrow*) (D). The *arrowheads* indicate the parasitic lesions in the liver of infected mice (“lesion” row). Final magnification, 400×. ^*^ *P*<0.05 versus control. con, control, non-infected mice; Em, *E. multilocularis* infected mice; AU: arbitrary units; lesion: *E. multilocularis* metacestode and surrounding immune infiltrate; close: liver parenchyma close to *E. multilocularis* lesion.

As shown in Figure 3A and 3B, western blotting showed increased Gadd45β expression from day60 (∼1.75-fold) to 360 (∼2.52-fold); it peaked at day180 (∼62.64-fold) post*-*infection; there was a significant difference between *E. multilocularis-*infected and non-infected mice at days 60 and 180 (*P* < 0.05). Gadd45β mRNA expression was increased from day2 (∼1.27-fold) to 360 (∼1.81-fold); it peaked at day 90 (∼3.28-fold) post*-*infection (Figure 3C). There was a significant difference between *E. multilocularis* infected and non-infected mice at days 60 and 90 (*P* < 0.05). At day8, Gadd45β expression was observed in infiltrating lymphocytes; it progressively increased in fibroblast-like cells and infiltrating lymphocytes from day30 to 180 (Figure 3D). Within the liver parenchyma close to the lesion and peri-parasitic infiltrate, Gadd45β expression was observed in hepatocytes around the portal and central veins at day30; it markedly increased at days 90 and 180 post-infection, with staining intensities from “moderate” to “strong”; then Gadd45β expression in hepatocytes mildly decreased at days 270 and 360. No liver cell positive for Gadd45β was observed in the livers of non-infected mice.

### Expression of Cyclins A, B1, D1 and E in the liver of mice during *E. multilocularis* infection

**Figure 4:**
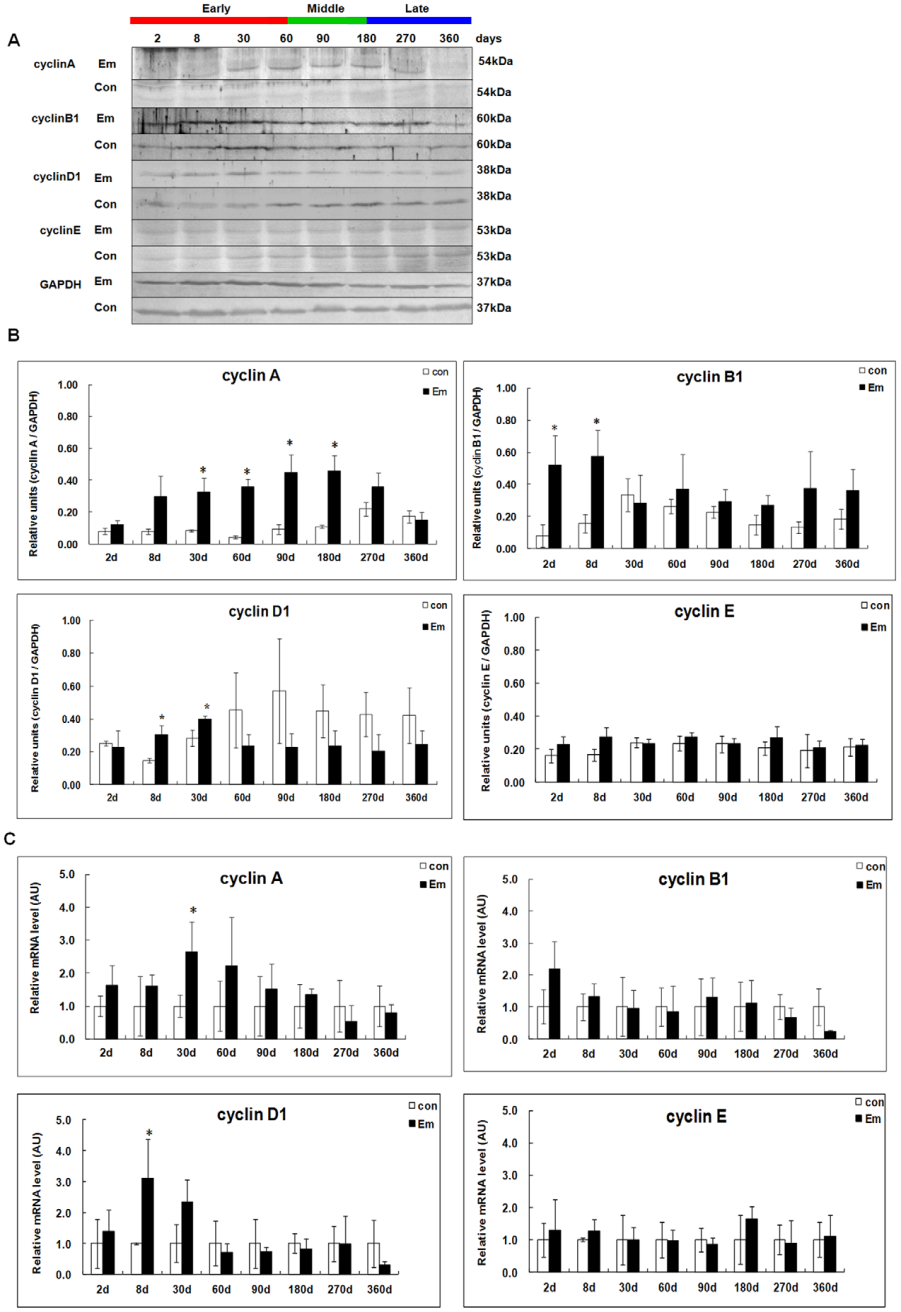
Cyclins A, B1, D1 and E expression in the liver of mice during *E. multilocularis* infection. Western blot analyses were performed on lysates with antibodies that recognize cyclins A, B1, D1 and E (A). Relative amount of cyclins A, B1, D1 and E expression was calculated from semi-quantitative analysis of the Western blots using densitometry (B). Cyclins A, B1, D1 and E mRNA expression was measured in the liver from *E. multilocularis* infected or non-infected mice by qPCR(C). ^*^ *P*<0.05 versus control. con, control, non-infected mice; Em, *E. multilocularis* infected mice; AU: arbitrary units.

As shown in Figure 4A and 4B, western blotting showed increased Cyclin A expression from day2 (∼1.52-fold) to 270 (∼1.63-fold); it peaked at day60 (∼8.46-fold), and then decreased under the baseline at day360 (∼0.86-fold) post*-*infection. There was a significant difference between *E. multilocularis* infected and non-infected mice at days 8, 30, 60, 90 and 180 (*P* < 0.05). Increased Cyclin B1 expression was observed at days 2 (∼6.53-fold) and 8 (∼3.71-fold); it then decreased to the baseline from day 30 to 360 post*-*infection. There was a significant difference between *E. multilocularis* infected and non-infected mice at days 2 and 8 (*P* < 0.05). Increased Cyclin D1 expression was observed at days 8 (∼2.07-fold) and 30 (∼1.41-fold); it then decreased under the baseline from day60 to 360 post*-*infection. There was a significant difference between *E. multilocularis* infected and non-infected mice at days 8 and 30 (*P* < 0.05). Mild increase in Cyclin E expression was observed from day 2 (∼1.44-fold) to 180 (∼1.30-fold), but no significant difference was found during the whole time-points (*P* > 0.05).

Cyclin A mRNA expression was increased from day2 (∼1.62-fold) to 180 (∼1.35-fold) and then decreased under the baseline at days 270 and 360; it peaked at day30 (∼2.65-fold) post*-*infection (Figure 4C). There was a significant difference between *E. multilocularis* infected and non-infected mice at day30 (*P* < 0.05). Cyclin B1 mRNA expression was increased at day 2 (∼2.17-fold); it then decreased to the baseline from day30 to 360 post*-*infection. However, there was no significant difference between *E. multilocularis* infected and non-infected mice (*P* > 0.05). Cyclin D1 mRNA expression was increased from day2 (∼1.41-fold) to 30 (∼2.34-fold); then it decreased under the baseline from day 60 to 360 post*-*infection. There was a significant difference between *E. multilocularis* infected and non-infected mice at day8 (*P* < 0.05). Cyclin E mRNA expression was mildly increased at days 2 (∼1.30-fold), 8 (∼1.25-fold) and 180 (∼1.64-fold), but no significant difference was found during the whole course of infection (*P* > 0.05).

### Expression of PCNA in the liver of mice during *E. multilocularis* infection

**Figure 5:**
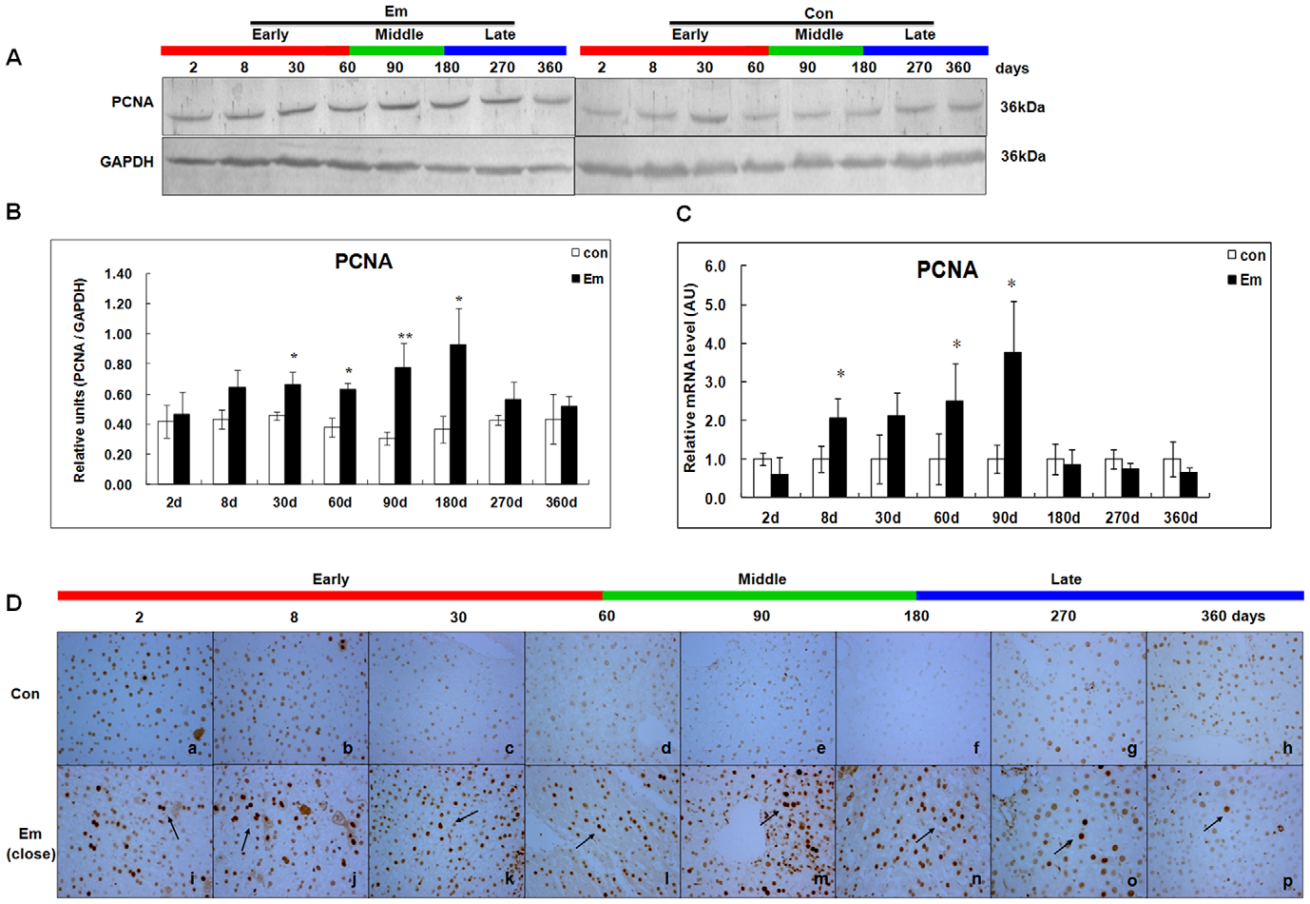
PCNA expression in the liver of mice during *E. multilocularis* infection. Western blot analyses were performed on lysates with antibodies that recognize PCNA (A). Relative amount of PCNA expression was calculated from semi-quantitative analysis of the Western blots using densitometry (B). PCNA mRNA expression was measured by qPCR (C) and PCNA expression by hepatic cells (close to lesion) in the liver from *E. multilocularis* infected or non-infected mice by histo-immunochemical analysis(*arrow*) (D). Final magnification, 400×. ^*^ *P*<0.05 versus control. con, control, non-infected mice; Em, *E. multilocularis* infected mice; AU: arbitrary units.

As shown in Figure 5A and 5B, western blotting showed increased PCNA expression from day8 (∼1.49-fold) to 180 (∼2.52-fold) which peaked at day90 (∼2.54-fold) post*-*infection; it then decreased to the baseline at days 270 (∼1.33-fold) and 360 (∼1.19-fold). There was a significant difference between *E. multilocularis-*infected and non-infected mice at days 30, 60, 90 and 180 (*P* < 0.05). PCNA mRNA expression was increased from day8 (∼2.06-fold) to day90 (∼3.74-fold); it peaked at day90 (Figure 5C), then decreased to the baseline from day180 to 360. There was a significant difference between *E. multilocularis* infected and non-infected mice at days 8, 60 and 90 post-infection (*P* < 0.05). As shown in Figure 5D, immunohistochemistry showed an increased expression of PCNA in the hepatocytes of *E. multilocularis* infected and non-infected mice at days 2 and 8, then no PCNA expression was observed in the liver of non-infected mice; increased expression of PCNA was observed since day 8 until day180 in infected mice.

### Expression of p53 in the liver of mice during *E. multilocularis* infection

**Figure 6:**
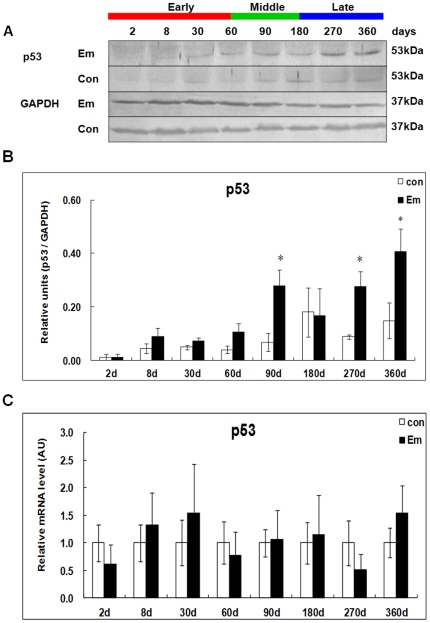
p53 expression in the liver of mice during *E. multilocularis* infection. Western blot analyses were performed on lysates from liver samples with antibodies that recognize p53 (A). Relative amount of p53 expression was calculated from semi-quantitative analysis of the Western blots using densitometry (B). p53 mRNA expression was measured in the liver from *E. multilocularis*-infected or non-infected mice by qPCR (C). ^*^ *P*<0.05 *versus* control. con, control, non-infected mice; Em, *E. multilocularis* infected mice; AU: arbitrary units.

As shown in Figure 6A and 6B, western blotting showed increased p53 expression at days 60 (∼2.63-fold), 90 (∼4.11-fold), 270 (∼3.13-fold) and 360 (∼2.77-fold); it peaked at day360 post*-*infection. There was a significant difference between *E. multilocularis*-infected and non-infected mice at days 90, 270 and 360 (*P* < 0.05). As shown in Figure 6C, no significant change of p53 mRNA expression was observed at any time-point.

### Expression of p21 in the liver of mice during *E. multilocularis* infection

**Figure 7:**
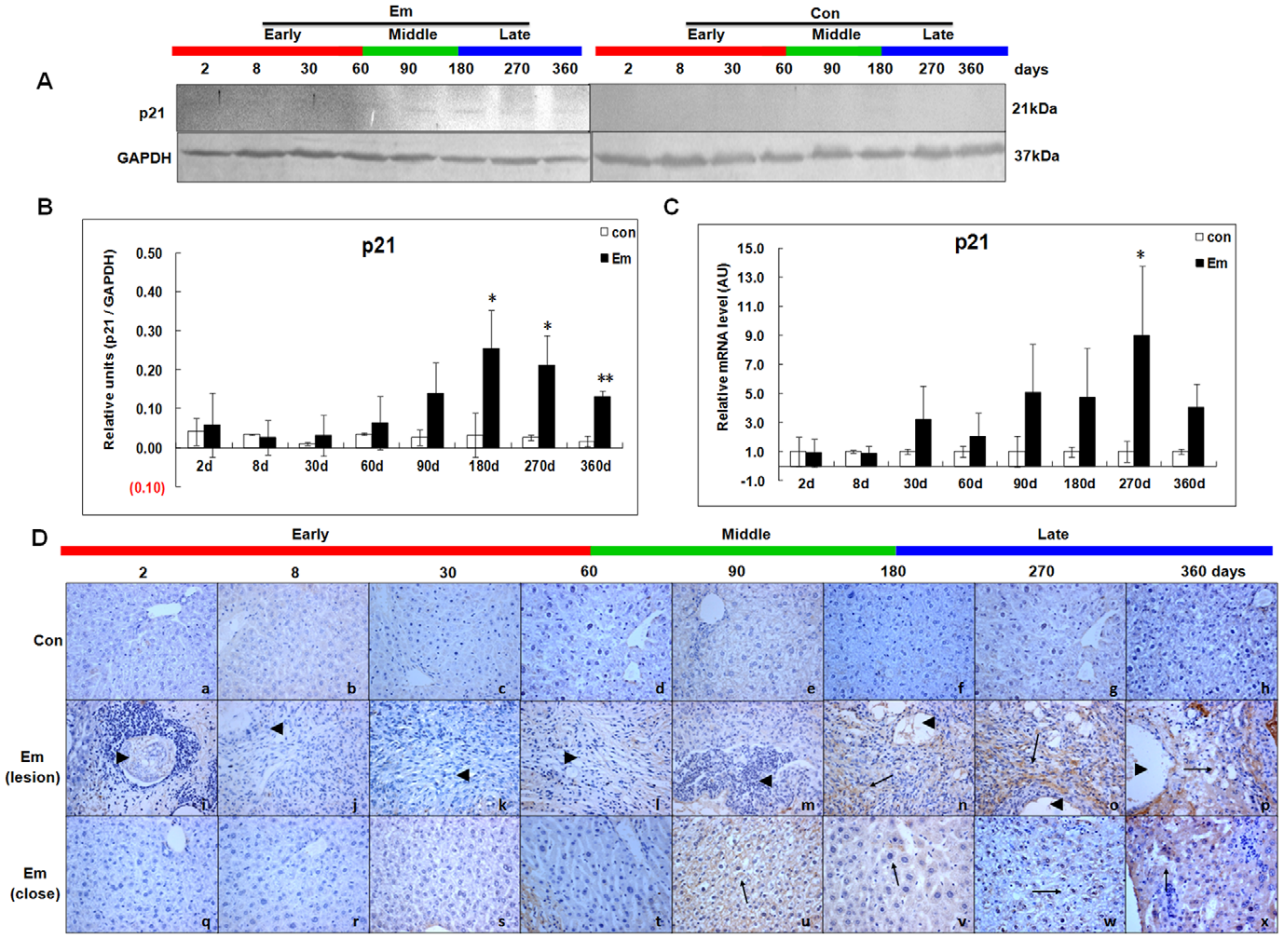
p21 expression in the liver of mice during *E. multilocularis* infection. Western blot analyses were performed on lysates from liver samples with antibodies that recognize p21 (A). Relative amount of p21 expression was calculated from semi-quantitative analysis of the Western blots using densitometry (B). p21 mRNA expression was measured by qPCR (C). Histo-immunochemical analysis of p21 expression was performed on tissue samples: p21 expression was observed in infiltrating lymphocytes (lesion) and fibroblast-like cells (lesion) but also in hepatic cells (close to lesion) in the liver from *E. multilocularis* infected mice (*arrow*) (D). The *arrowheads* indicate the parasitic lesions in the liver of infected mice (“lesion” row). Final magnification, 400×. ^*^ *P*<0.05 *versus* control. con, control, non-infected mice; Em, *E. multilocularis* infected mice; AU: arbitrary units; lesion, *E. multilocularis* metacestode and surrounding immune infiltrate; close, liver parenchyma close to lesion.

As shown in Figure 7A and 7B, western blotting showed increased p21 expression from day60 (∼1.84-fold) to 360 (∼8.15-fold); it peaked at day360 post*-*infection. The difference between *E. multilocularis*-infected and non-infected mice was significant at days 180, 270 and 360 (*P* < 0.05). As shown in Figure 7C, p21 mRNA expression, despite its increase from day30 (∼3.21-fold) to 360 (∼4.06-fold), was significantly different from that measured in non-infected mice when it peaked at day270 post-infection (∼9.01-fold) (*P* < 0.05). As shown in Figure 7D, in the lesion area, p21 expression was observed in infiltrating lymphocytes and fibroblast-like cells at day180; it progressively increased from day270 to 360. Within the liver parenchyma close to the lesion and peri-parasitic infiltrate, p21 expression was observed in hepatocytes close to the lesion at day180. A marked increase in the liver cell expression of p21 was observed at days 270 and 360 post-infection, with staining intensities from “weak” to “moderate”.

### Expression of Gadd45γ in the liver of mice during *E. multilocularis* infection

**Figure 8:**
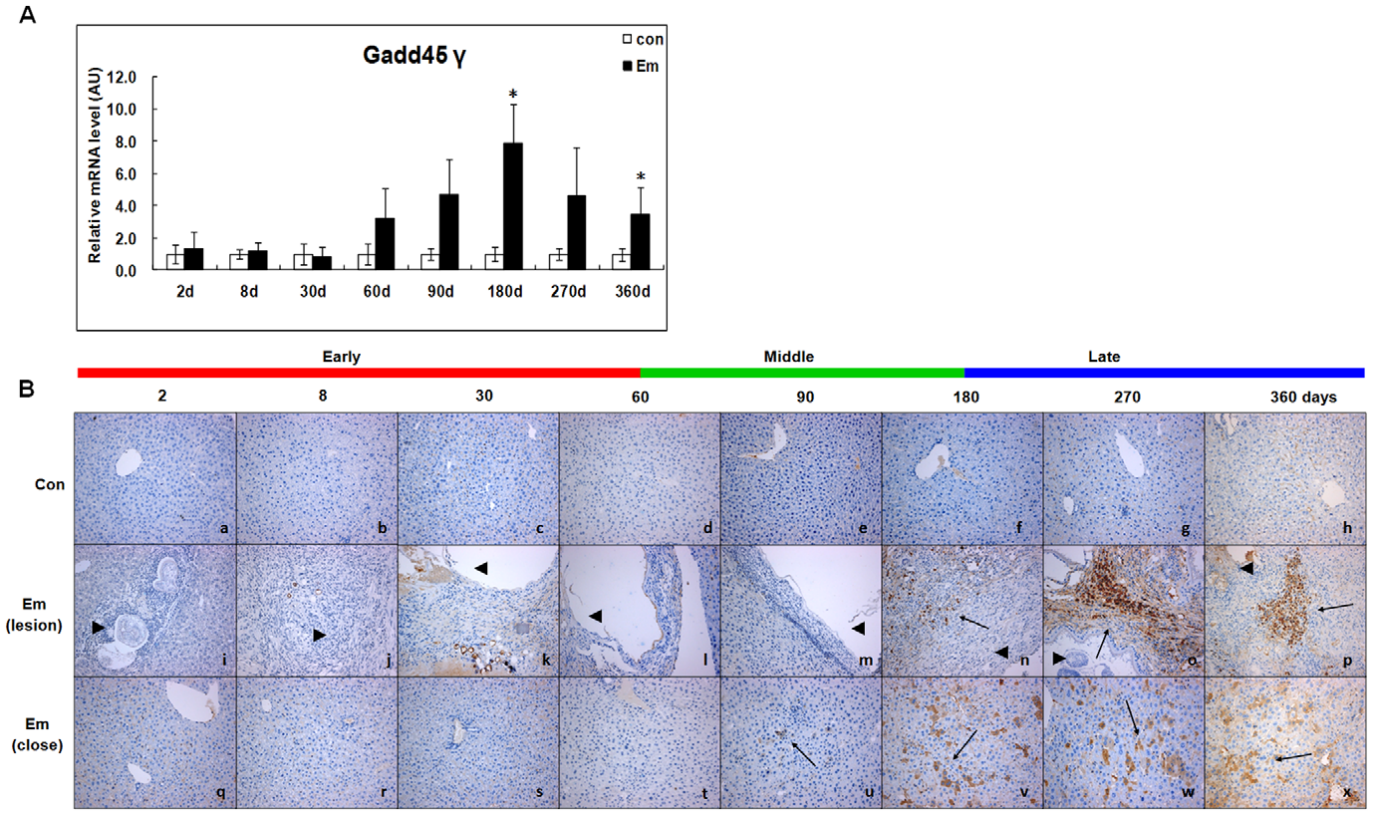
Gadd45γ expression in the liver of mice during *E. multilocularis* infection. Gadd45γ mRNA expression was measured by qPCR (A). Histo-immunochemical analysis of Gadd45γ expression was performed on tissue samples. Gadd45γ expression was observed in infiltrating lymphocytes (lesion) and hepatic cells (close to lesion) in the liver from *E. multilocularis*-infected or non-infected mice (*arrow*) (B). The *arrowheads* indicate the parasitic lesions in the liver of infected mice (“lesion” row). Final magnification, 200×. ^*^ *P*<0.05 *versus* control. con, control, non-infected mice; Em, *E. multilocularis* infected mice; AU: arbitrary units; lesion, *E. multilocularis* metacestode and surrounding immune infiltrate; close, liver parenchyma close to *E. multilocularis* lesion.

As shown in Figure 8A, before day60, Gadd45γ mRNA expression was unchanged; it then increased from day60 (∼3.23-fold) to 360 (∼3.47-fold) and peaked at day180 (∼7.86-fold) post*-*infection; it was significantly different between *E. multilocularis*-infected and non-infected mice at days 180 and 360 post-infection (*P* < 0.05). As shown in Figure 8B, Gadd45γ expression was observed in periparasitic infiltrating lymphocytes at day180; it increased inside the lesion at days 270 and 360. Within the liver parenchyma close to the lesion and peri-parasitic infiltrate, Gadd45γ expression was observed at day90 and markedly increased at days 180, 270 and 360 post-infection, with staining intensities from “moderate” to “strong”. No liver cell positive for Gadd45γ was observed in the livers of non-infected mice.

### Activation of caspase 3 in the liver of mice during *E. multilocularis* infection

**Figure 9:**
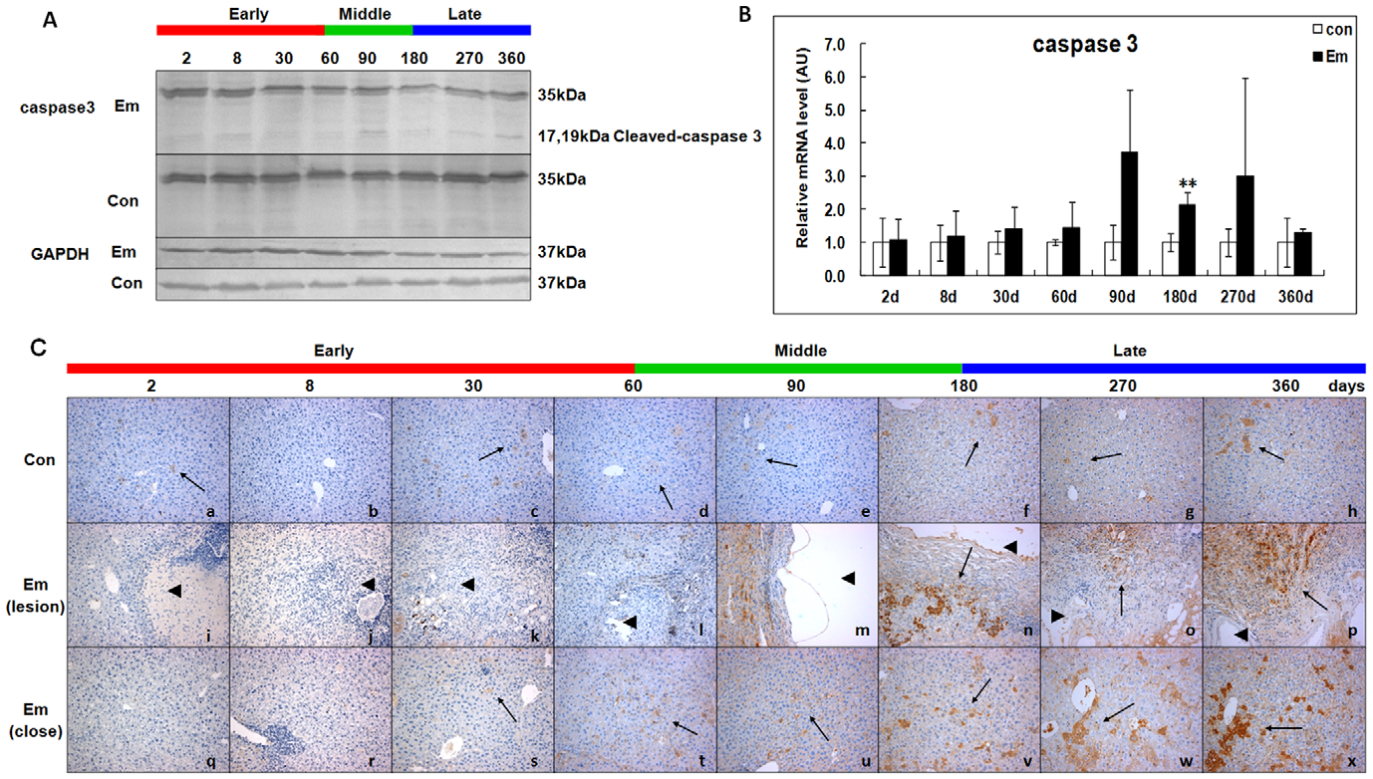
Activation of caspase 3 in the liver of mice during *E. multilocularis* infection. Western blot analyses were performed on lysates from liver samples with antibodies that recognize caspase 3 and cleaved-caspase 3 (A). Caspase 3 mRNA expression was measured by qPCR (B). Histo-immunochemical analysis of caspase 3 and cleaved-caspase 3 expression was performed on tissue samples. Caspase 3 and cleaved-caspase 3 expression was observed in infiltrating lymphocytes (lesion) and hepatic cells (close to lesion) in the liver from *E. multilocularis*-infected or non-infected mice (*arrow*) (C). The *arrowheads* indicate the parasitic lesions in the liver of infected mice (“lesion” row). Final magnification, 200×. ^**^ *P*<0.01 *versus* control. con, control, non-infected mice; Em, *E. multilocularis* infected mice; AU: arbitrary units; lesion, *E. multilocularis* metacestode and surrounding immune infiltrate; close, liver parenchyma close to *E. multilocularis* lesion.

Caspase 3 activation, a marker of the apoptotic protease cascade, was measured by western blotting, qPCR and immunohistochemistry. As shown in Figure 9A which shows immunoblotting with anti-caspase 3 (35kDα) and anti-cleaved caspase 3 (17 and 19kDα), cleaved caspase 3 was significantly increased at days 90, 270 and 360 post*-*infection. As shown in Figure 9B, before day60, caspase 3 mRNA level was unchanged; it then increased from day60 (∼1.43-fold) to 270 (∼2.98-fold), peaked at day90 (∼3.70-fold), and decreased to the baseline at day360. There was a significant difference between *E. multilocularis*-infected and non-infected mice at day180 post-infection (*P* < 0.01). As shown in Figure 9C, in the periparasitic infiltrate, caspase 3 and cleaved-caspase 3 expression were observed in the lymphocytes at day90; it then increased inside the lesion from day180 to 360. Within the liver parenchyma close to the lesion and peri-parasitic infiltrate, caspase 3 and cleaved-caspase 3 expression was observed at days 60 and 90; it then markedly increased at days 270 and 360 post-infection, with staining intensities from “moderate” to “strong”. Minimal immunostaining for caspase 3 and cleaved-caspase 3 was observed in the livers of non-infected mice.

### TUNEL-positive cells in the liver of mice during *E. multilocularis* infection

**Figure 10:**
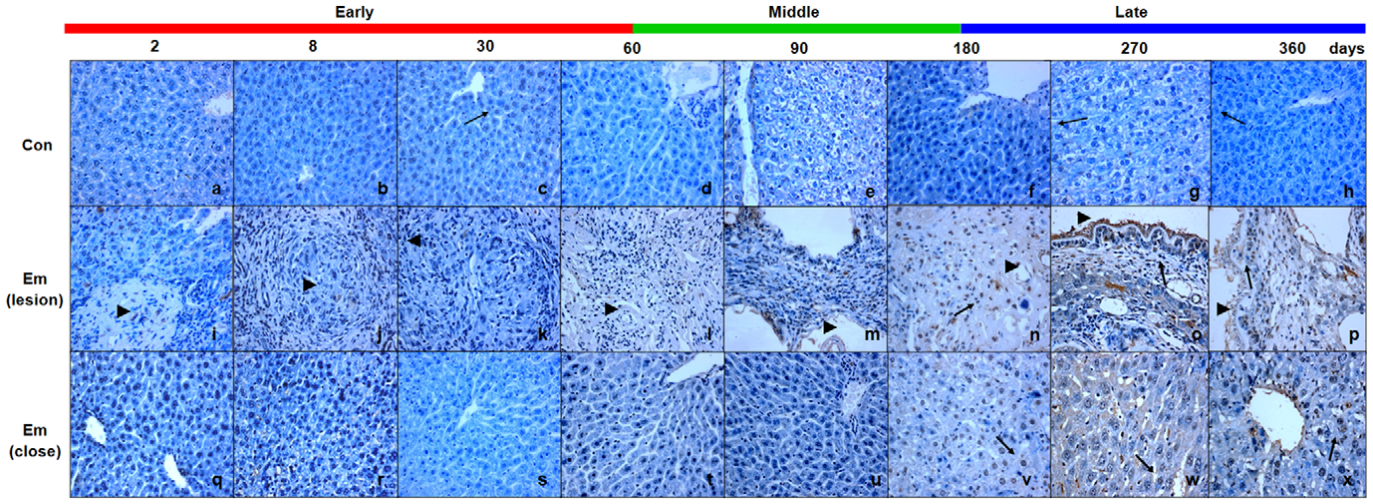
TUNEL assay in the liver during *E. multilocularis* infection. TUNEL assays were performed on the livers from *E. multiloculari*-infected and non-infected mice. Some TUNEL-positive infiltrating lymphocytes (lesion) and hepatic cells(close to lesion) were found in the liver of infected mice from day180 to 360; None was found at day60 and 90 (*arrows*). The *arrowheads* indicate the parasitic lesions in the liver of infected mice (“lesion” row). No TUNEL-positive cells were seen in the liver of non-infected mice. Final magnification, 400×. con, control, non-infected mice; Em, *E. multilocularis* infected mice; AU: arbitrary units; lesion: *E. multilocularis* metacestode and surrounding immune infiltrate; close: liver parenchyma close to *E. multilocularis* lesion.

To determine whether cells exhibited DNA strand breakage during *E.multilocularis* infection, and could thus be considered apoptotic, the TUNEL assay was applied to the liver sections. As shown in Figure 10, in the periparasitic infiltrate, some inflammatory cells with apoptosis were observed inside the lesion at days 180, 270 and 360. Within the liver parenchyma close to the lesion and peri-parasitic infiltrate, a few hepatocytes exhibiting apoptosis were observed at day180; the number of apoptotic hepatocytes significantly increased in a time-dependent manner up to day360 post-infection; during the late stage of infection, TUNEL staining intensities ranged from “weak” to “moderate”. No apoptotic liver cells were observed in the livers of the non-infected mice.

## Discussion

**Figure 11:**
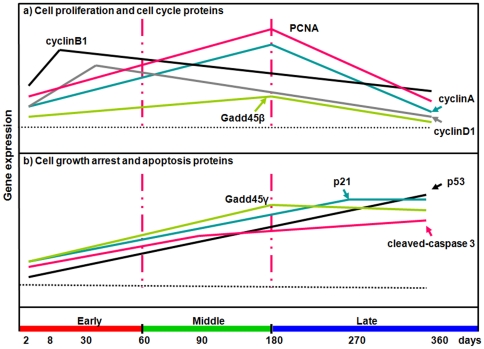
Overall changes in the gene expression of cell proliferation/cell cycle proteins (a) and cell growth arrest/apoptosis proteins (b) during the process of *E. multilocularis*-induced liver injury in mice.

Changes in the metabolic pathways involved in the regulation of hepatic cell proliferation and growth arrest, and especially in the MAPKs system, have been extensively studied in infectious/inflammatory conditions, especially of viral origin [36], [37], [38]. Very little is known of the influence of helminth parasites which develop in the liver on the proliferation/growth arrest of the hepatocytes in the infected liver. Until recently, no study had ever specifically addressed the issue of liver proliferation/regeneration and growth arrest/apoptosis after *E. multilocularis* infection. In this longitudinal study using the murine experimental model of *E. multilocularis* infection, we could show that, opposite to those involved in cell proliferation and anti-apoptosis which were activated in the first half of the infection course, metabolic pathways involved in growth arrest and apoptosis were significantly activated in the liver of the infected mice in the second half of the infection course (Figure 11).

In the present study, we could confirm the induction of p-ERK1/2 and a parallel expression of Cyclin D1 and PCNA from the beginning of infection and their persistence during the progression of *E. multilocularis* growth in the liver during the first 2 stages of parasite development. This suggested that, as shown in other models, activation of the ERK1/2 pathway increased hepatocyte DNA synthesis and was involved in the activation of cell cycle [39], [40], [41]. Increased expression of PCNA in the hepatic cells under the influence of *E. multilocularis* was constantly found *in vivo*, in previous studies [33], [34] and in this study as well. PCNA is a stable cell cycle-related 36 kDa nuclear protein which is increasingly expressed in late G1 and throughout S phase of the cell cycle. Its rate of synthesis is correlated with the proliferative rate of cells, and PCNA immunoblot and immunostaining can be used to reliably define and map proliferating cells in animal and human tissues [42], [43]. Expression of PCNA in the liver hepatocytes of both infected and non-infected mice at days 2 and 8 after infection clearly shows the regeneration process induced by the injection in the liver, which constitutes, per se, a liver injury. In our study, qPCR confirmed that intrahepatic PCNA synthesis was more increased in *E. multilocularis*-infected mice than in non-infected mice as early as day8, and that PCNA synthesis increase was also correlated with *E. multilocularis* development until day180. Then we also found that the induction of p-JNK induced by metacestode components we observed *in vitro* in a previous study [33] began at the end of the middle stage of infection and remained markedly increased at its late stage (up to 1 year). Such activation paralleled an up-regulation of p53, p21, Gadd45γ, an increased expression of cleaved-caspase 3, an increase in the number of TUNEL-positive (apoptotic) cells in the lesion and in the liver parenchyma, and a down-regulation of PCNA and Cyclin A. Activation of the metabolic pathways which govern growth arrest and apoptosis also paralleled the previously described decrease of lymphocyte proliferation and of cytokine production observed at the late stage of experimental infection [10], [11], [12].

Accumulated data have suggested that the stress- and cytokine-inducible Gadd45 family proteins (Gadd45α, Gadd45β, Gadd45γ) serve similar but not identical functions along various pro- or anti-apoptotic and growth-suppressive pathways [44]. Gadd45β, which interacts with critical cell cycle regulatory proteins, such as PCNA, Cdk1 and Cyclin B1, plays an active role in cell cycle adjustment [27], [35], [45]. From our previous microarray analysis, we could observe Gadd45β up-regulation at days 90 and 180 in the murine AE model [34], which highly suggested that up-regulated expression of Gadd45β gene might actually be a trigger for hepatic cell survival. Our present study showed that Gadd45β mRNA was significantly increased from day60 to 180. Both immunostaining and immunoblotting confirmed that Gadd45β protein was actually expressed in the liver during the same period, especially in the vicinity of the metacestode. Interestingly, Gadd45β is able to inhibit TNF-α-induced cytotoxicity and Fas-induced apoptosis [19], [46], [47], both of which were showed to be expressed at the periphery of the periparasitic granuloma, at the border of the liver parenchyma, in human AE [3], [48]. A recent study has demonstrated that ERK and Gadd45β are closely related to NF-κB activity, forming a loop-like connection to increase cell survival after lethal damage induced by ionizing radiation [49]. Taken together, these observations suggest that activation of ERK1/2, Gadd45β and Cyclin A are able to interact and promote hepatocyte anti-apoptosis at the middle stage of infection. Gadd45γ is a critical and essential mediator of apoptosis induction among the three Gadd45 isoforms because Gadd45γ has strongly pro-apoptotic roles at all times [50]. Gadd45γ may interact with MTK1/MEKK4, which in turn activate both JNK and p38 leading to apoptosis in response to environmental stresses [51], [52], [53]. Our data obtained during the course of infection suggested a sequential activation of, first, Gadd45β in the first stages of infection, and, then, of Gadd45γ in the late stage of infection; however, the factors which are responsible for the shift to Gadd45γ expression after day180 of infection are unknown. Our present study showed that, like Gadd45γ expression, activation of JNK was observed from day180 to 360. Generally, JNK acts as a critical mediator of hypoxia/reoxygenation-induced apoptosis and also of TNF-induced apoptosis by glutathione depletion in hepatocytes [54], [55]. Our data also suggest that p53-dependent mechanisms (p53-p21 and p53-Gadd45γ signaling pathways) may be involved following *E. multilocularis* infection to induce hepatocyte growth arrest/apoptosis. Our data regarding TUNEL-positive cells and elevation of cleaved-caspase 3 highly suggest that hepatocyte apoptosis is a significant event at the late stage of infection. These observations are consistent with the results of previous studies which showed that apoptosis-mediated damage was present in other parasitic diseases such as Chagas^'^ disease, toxoplasmosis, leishmaniasis, schistosomiasis due to *Schistosoma mansoni*, and cerebral malaria [56], [57], [58], [59], [60]. Hepatocyte apoptosis at the late stage of infection could result from either toxic by-products originating from the parasite or from parasite-induced inflammation. But unlike growth arrest/apoptosis, the activation of hepatocyte proliferation/anti-apoptosis processes we observed in the same experimental mice appears to be a less common phenomenon, which could represent one of the specificities of *E. multilocularis* infection at its early stages.

**Figure 12:**
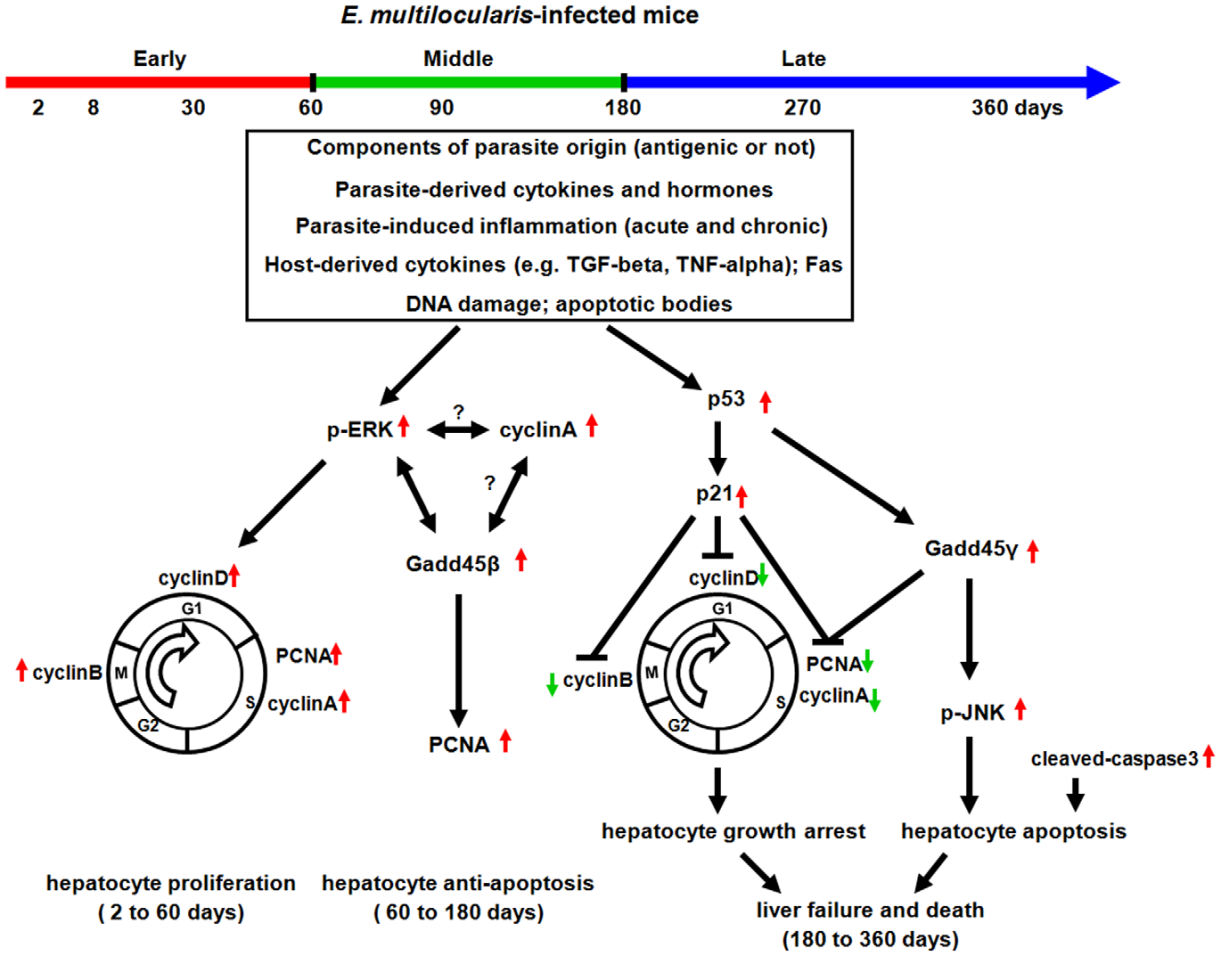
Schematic diagram summarizing the metabolic pathways involved in hepatocyte proliferation/anti-apoptosis and growth arrest/apoptosis in the liver of mice with *E. multilocularis* infection.

Hepatocytes suffering sublethal injury have the capacity to activate an internally-triggered cell regeneration mechanism and our previous studies as well as this one have brought evidence that, as it also occurs during viral infection or toxic injury [61], this regeneration mechanism was also operating in *E. multilocularis* infection [33], [34], and was especially prominent at the first stages of infection, as was shown in experimental mice until day180 after infection. Liver regeneration is controlled by a wide array of signaling factors and plays a key role in recovery after acute and chronic liver injury. Hepatic cell proliferation is essential to enhance or restore hepatic function [62], [63]. Although hepatocyte proliferation is often mediated by the injury/regeneration response, however, in other circumstances it is part of an adaptive response to stress stimuli which do not lead to cell death (direct hyperplasia). This proliferative response is regulated by cell cycle regulated proteins [64]. In AE, influence of the parasite on hepatocyte proliferation (and/or anti-apoptosis) is supported by the up-regulation of Cyclins A, B1, D1 and Gadd45β, which is summarized in Figure 10. Until day180, i.e. in the early and middle stages of infection, gene expression level of CyclinA was increased in a time-dependent manner, while gene expression levels of Cyclin B1 and CyclinD1 were increased up to day30 and then returned to the control level after day60. On the other hand, no significant change in the expression of Cyclin E was observed at any time during the period of observation. Up-regulation of PCNA, Cyclin D1, A and B1 is related to the regulation of the G1/S and G2/M phases [65], [66], which were previously reported to increase biphasically after partial hepatectomy and other parasitic infection [67], [68]. The “late stage” of infection, i.e. after day180 after infection, has rarely been studied in the murine model of secondary (or primary) *E. multilocularis* infection. In the most susceptible mice, impairment of vital functions due to *E. multilocularis* progression and metastases is fast and occurs between day180 and 270, which makes studies difficult to interpret. In addition, most of the studies addressed immunological mechanisms of immune tolerance; since they were just failing at that late stage [10], [11], [12], it was thus considered of less interest for that purpose. As the experimental mice we are working with, albeit quite susceptible to *E. multilocularis*, have a prolonged survival until day360, and because we observed activation of both proliferation and apoptosis pathways at day180, we decided to measure the expression and/or activation of the components of these pathways until day360. We were thus able to show the mirror image of growth arrest/apoptosis *versus* proliferation/anti-apoptosis during the natural course of metacestode progression in the liver (Figure 12). These results might suggest that the proliferative capability of hepatocytes was exhausted during continuously lasting hepatic damage, due to direct toxicity of parasitic components and/or cytotoxic attacks by the immune system. This exhaustion might also be due to the profound malnutrition (wasting disease/cachexia) observed in *E. multilocularis*-infected mice in the advanced stage of the disease, and the altered ability of liver cells to synthesize proteins, as suggested by the changes in the expression of many genes at this stage in the microarray analysis we recently performed [34] . However, during the early and middle stage of infection, despite the presence of the metacestode and its growth, very little necrosis is observed on the liver pathological sections in the experimental model; we could confirm such observations [69]. Necrosis of the liver lesion is not observed in all patients with AE: it has only been observed in more advanced/severe cases, and was associated with susceptibility markers and/or with expression of TNF-α by the periparasitic macrophages [48]. On the other hand, the immune tolerance generated by the presence of *E. multilocularis* metacestode in the liver is associated with a poor development of NK cytotoxicity and an inhibition of T-lymphocyte-dependent cytotoxicity, despite the high proliferation potential of T-lymphocytes, the presence of numerous CD8 T-lymphocytes in the liver within the parasitic lesion, and the expression of the appropriate ligands, such as MICA/B [70], [71], [72]. Such an inhibition is possibly due to the combined influence of IL-10 and TGF-β production [7], [73], [74]. In addition, hepatocytes do not die but do proliferate in response to parasite growth at the early and middle stages of infection, and we could observe a significant increase in the expression and/or activation of the components of the proliferation pathways by hepatic cells at that stage. Such observations at least partially rule out a direct influence of cytotoxic components, which would manifest itself at all stages of parasite development. Similar “exhaustion” at the late stage of the disease has also been observed for lymphocyte proliferation and cytokine secretion in susceptible experimental mice [10], [11], [12]. In patients with AE, incubation of peripheral blood mononuclear cells with parasitic antigens has been shown to induce significantly less proliferation in AE patients with severe disease than in patients with abortive or surgically cured cases [75]: taken together, these observations suggest a common negative influence of parasitic components on all types of cell proliferation, which would become patent only at an advanced stage of *E. multilocularis* development.

From day90 to 180 after infection, in the BALB/c mice, AE vesicles have become fully fertile and exhibit a number of protoscoleces; it might be hypothesized that this period represents the critical phase when the maturation of the metacestode has been achieved and after which the host is no longer required to promote parasite growth and fertility. In our experiments, day90 and 180 represented the critical steps when we still observed expression of the proliferation pathway components and peaks in the expression/activation of anti-apoptotic pathway components, as well as beginning of increase in the expression/activation of growth arrest/apoptotic pathway components. Then, growth arrest/apoptotic pathways clearly overcome proliferation/anti-apoptotic pathways. Our previous *in vitro* experiments showed that such sequential events were not associated with any non-specific “exhaustion”, which could only be observed *in vivo*. They strongly suggested that they are governed by components of parasitic origin: activation of such pathways was observed in isolated hepatocytes under the influence of components present in the vesicle fluid and in the supernatants of metacestode axenic cultures [33]. Future work should elucidate the nature of these components which would act in a sequential manner: 1) at the early stage and beginning of the middle stage of *E. multilocularis* infection, they would favor parasitic cell, liver cell and immune cell proliferation and survival, and promote metacestode fertility and tolerance by the host, and 2) after fertility has been acquired, at the end of middle stage and all late stage of infection, they would contribute to the dissemination of the protoscoleces and next steps of the parasite cycle by favoring liver damage/apoptosis and subsequent major impairment in protein synthesis and xenobiotic metabolism [34], as well as immune deficiency [10], [11], [12], [75], and eventually lead to the host's death.

In conclusion, this study is the first report which demonstrates a coordinated activation of the proliferation/anti-apoptosis and growth arrest/apoptosis mechanisms *in vivo* during the course of *E. multilocularis* infection. As suggested in a schematic diagram (Figure 12): 1) at the early stage of infection, activation of ERK1/2 and downstream targets such as CyclinD1, A, B1 and PCNA would favor hepatocyte proliferation, 2) at the middle stage of infection, permanent activation of ERK1/2 and Cyclin A and elevated Gadd45β expression may synergize to mediate an anti-apoptotic response to enhance liver cell survival and prevent lethal tissue damage induced both by the parasite itself and cytokines such as TNF-α, and 3) at the late stage of infection, activation of JNK and the increased expression of p53, p21, Gadd45γ and cleaved-caspase 3 would induce hepatocyte growth arrest/apoptosis. These findings also give a rational explanation to the clinical observations: 1) hepatomegaly of unusual size is frequent at presentation; and an unexpected survival rate is observed after major hepatic resection in AE patients [76], and 2) chronic liver injury, necrosis, and hepatic failure in AE patients at an advanced stage and in experimental animals [6], [9]. They provide a molecular basis for the balance between liver damage and repair in the progression of *E. multilocularis* infection.

## Materials and Methods

### Ethics Statement

All animals received humane care in compliance with the Medical Research Center's guidelines, and animal procedures were approved by the Animal Care and Use Committee and the Ethical Committee of First Affiliated Hospital of Xinjiang Medical University (20081205-2).

### Experimental design

Pathogen-free female BALB/c mice (8–10 weeks old) were purchased from the Animal Center of Xinjiang Medical University and were maintained in an air-conditioned animal room with a 12-h light/dark cycle and provided with rodent chow and water. *E*. *multilocularis* metacestodes were obtained from intraperitoneal lesions maintained in *Meriones unguiculatus*, and 0.1 mL of pooled lesions (∼1, 000 protoscoleces) was injected into the anterior liver lobe of infected mice as previously described [69]. For each autopsy time-point, ten experimentally infected mice were used in *E*. *multilocularis* group (n = 10) and compared with five control mice (n = 5), which received an intra-hepatic injection of 0.1 mL of saline in the anterior liver lobe using the same surgical procedure. Mice were killed at days 2, 8, 30, 60, 90, 180, 270 and 360.

### Tissue sampling

In *E. multilocularis* infected mice, liver tissue samples were taken close to the parasitic lesions, i.e. 1–2mm from the macroscopic changes due to the metacestode/granuloma lesion (white-yellowish color), thus avoiding liver contamination with parasitic tissue and infiltrating immune cells; in control mice, liver tissue samples were taken from the same (anterior) liver lobe in the sham-injected area. Tissue fragments were separated into two parts and either deep-frozen in liquid nitrogen for western blot and qPCR or formalin-fixed for immunohistochemistry. In addition, liver tissue samples were also taken from the parasitic lesion (including periparasitic liver tissue adjacent by 1 mm to the macroscopically visible parasitic lesion) for histopathology and immunohistochemistry.

### Pathology and parasitology

For histopathology livers were placed in 10% buffered formalin and then embedded in paraffin. Five micrometer thick microtome sections were prepared from each liver sample and stained with hematoxylin-eosin. The sections were examined for the pathological changes generated by *E. multilocularis* and for hepatotoxicity. The histological slides were evaluated blindly by two of the authors.

### Western blot analysis

Western blot analysis of cell lysates was performed by SDS-PAGE using NuPAGE (Invitrogen, California, USA) followed by transfer to PVDF membrane. Using the appropriate antibodies: against total and p-ERK1/2, p-JNK, p-38 (dilution 1∶500, Cell Signaling technology, Massachusetts, USA), Gadd45β (dilution 1∶800), Cyclins A, B1, D1 and E (dilution 1∶500), PCNA (dilution 1∶1000), p53, p21 (dilution 1∶500, Santa Cruz, California, USA), full length caspase 3 (35kDa) and large fragment of caspase 3 resulting from cleavage (17kDa) (dilution 1∶500, Cell Signaling technology, Massachusetts, USA), and GAPDH (dilution 1∶1000, Santa Cruz, California, USA) were detected using the WesternBreeze Kit (Invitrogen, California, USA). The expression levels of respective proteins (in “relative units”) in non-infected mice and infected mice were quantified using Quantity One software (Bio-Rad, Hercules, CA), according to a previously described procedure [77].

### Quantitative real-time PCR analysis (qPCR)

After removing contaminated DNA from the isolated RNA using DNaseI (Fermentas, Vilnius, Lithuania), 1 µg of total RNA was reverse transcribed into cDNA in 20 µL reaction mixtures containing 200U of Moloney murine leukemia virus reverse transcriptase (MMLV, Promega, Madison, USA); 100 ng per reaction of oligo (dT) primers; and 0.5mM each of dNTPs, dATP, dCTP, dGTP, and dTTP. The reaction mixture was then incubated at 42°C for 1 hour and at 95°C for 5 min to deactivate the reverse transcriptase.

The qPCR was run in a thermocycler (iQ5 Bio-Rad, Hercules, CA) with the SYBR Green PCR premix (TaKaRa, Dalian, China) following the manufacturer's instructions. Thermo-cycling was performed in a final volume of 20 µL containing 2 µL cDNA and 10 pmol of each primer (Table 1). To normalize for gene expression, mRNA expression of the housekeeping gene beta-actin was measured. For every sample, both the housekeeping and the target genes were amplified in triplicate using the following cycle scheme: after initial denaturation of the samples at 95°C for 1 min, 40 cycles of 95°C for 5 s and 60°C (or other) for 30 s were performed. Fluorescence was measured in every cycle, and a melting curve was analyzed after the PCR by increasing the temperature from 55 to 95°C (0.5°C increments). A defined single peak was obtained for all amplicons, confirming the specificity of the amplification.

**Table 1:** Primers and cycling parameters of qRT-PCR.

Gene	Genbank accession	Primer Sequences	Annealing temperature	Expected Size
beta-actin	NM_007393	F: 5′-AACTCCATCATGAAGTGTGA-3′ R: 5′-ACTCCTGCTTGCTGATCCAC-3′	56°C	248bp
PCNA	NM_0110045	F: 5′- GAGAGCTTGGCAATGGGAACA-3′ R: 5′-GGGCACATCTGCAGACATACTGA-3′	60°C	185bp
CyclinA	NM_009828	F: 5′- AGCAGAAGAGACTCAGAAGAGG -3′ R: 5′- GATAGTCAAGAGGTGTCAGTGG -3′	60°C	121bp
CyclinB1	NM_172301	F: 5′- AGGAAGAGCAGTCAGTTAGACC -3′ R: 5′- CTGGAGGGTACATCTCCTCATA -3′	60°C	248bp
CyclinD1	NM_007631	F: 5′- GGGGACAACTCTTAAGTCTCAC -3′ R: 5′- CCAATAAAAGACCAATCTCTC -3′	60°C	206bp
CyclinE	NM_007633	F: 5′- GAGCTTGAATACCCTAGGACTG -3′ R: 5′-CGTCTCTCTGTGGAGCTTATAGAC -3′	60°C	245bp
Gadd45β	NM_008655	F: 5′- GAGGCGGCCAAACTGATGA-3′ R: 5′-TCGCAGCAGAACGACTGGA-3′	60°C	128bp
p53	NM_001127233	F: 5′- ATTGGGACCATCCTGGCTGTAG -3′ R: 5′- CGAGGCTGATATCCGACTGTGA -3′	60°C	148bp
p21	NM_007669	F: 5′-CTGTCTTGCACTCTGGTGTCTGA-3′ R: 5′-CCAATCTGCGCTTGGAGTGA-3′	60°C	121bp
Gadd45γ	NM_011817	F: 5′- TGGATAACTTGCTGTTCGTGGA-3′ R: 5′-CAGCAGAAGTTCGTGCAGTG-3′	60°C	122bp
Caspase 3	NM_009810	F: 5′- CTGCCGGAGTCTGACTGGAA-3′ R: 5′-ATCAGTCCCACTGTCTGTCTCAATG-3′	60°C	97bp

### Immunohistochemical staining

Paraffin-embedded liver tissue samples of control mice and infected mice were examined to determine the expression and distribution of p-ERK1/2, Gadd45β, PCNA, p21, Gadd45γ, caspase 3 and cleaved-caspase 3 protein at each time points. All sections were first deparaffinized and then incubated with 3% hydrogen peroxide for 10 min to block the endogenous peroxidase activity. After being washed with PBS and incubation for 1 hour with 5% normal goat serum to reduce non-specific background staining, the sections were incubated with the above mentioned primary antibodies against p-ERK1/2 (dilution 1∶50), Gadd45β (dilution 1∶100), PCNA (dilution 1∶300), p21 (dilution 1∶50), Gadd45γ (dilution 1∶200), caspase 3 and cleaved-caspase 3(dilution 1∶100) at 4°C overnight. Immunoreactive proteins were visualized using the appropriate anti-IgG secondary antibodies labeled with horseradish peroxidase (Santa Cruz, California, USA) and the chromogen 3′-diaminobenzidine (DAB, Zhongshan, Beijing, China) as a substrate[5]. Negative controls were incubated without primary antibodies but were otherwise subjected to all the immunohistochemical procedures. Sections were examined microscopically for specific staining and photographs were taken using a digital image-capture system (Leica, Solms, Germany); the intensity of positive hepatocyte staining was classified on an arbitrary scale including “negative”, “weak”, “moderate”, and “strong” staining.

### TUNEL analysis

Apoptosis in the livers of infected mice and non-infected mice was further analyzed using a commercial kit (Roche, Mannheim, Germany) based on the TdT-mediated dUTP-digoxigenin nick-end labeling (TUNEL) of apoptotic cells. Five micrometer sections of paraffin-embedded samples of liver were prepared as described above. Sections were examined microscopically for specific staining and photographs were taken using a digital image-capture system (Leica, Solms, Germany) (the intensity of TUNEL-positive hepatocyte staining was classed on an arbitrary scale from “negative” to “strong” staining).

### Statistical analysis

All data were presented as the means with standard deviation and analyzed using SPSS version 11.0 software (SPSS, Chicago, IL, USA). Statistical significance was tested using the Student's *t* test; a *P* value of less than 0.05 was considered significant.
